# Long-term surgical outcomes of basic-type exotropia in patients with hyperopia

**DOI:** 10.1186/s12886-023-02909-1

**Published:** 2023-04-27

**Authors:** Hyeshin Jeon, Hee-young Choi

**Affiliations:** 1 Department of Ophthalmology, School of Medicine, Pusan National University, Busan, South Korea; 2 Department of Ophthalmology and Medical Research Institute, Pusan National University Hospital, 1-10 Ami-Dong Seo-Gu, Busan, 47732 South Korea

**Keywords:** Exotropia, Hyperopia, Surgery

## Abstract

**Background:**

To investigate the surgical outcomes of basic-type exotropia in patients with hyperopia.

**Methods:**

The medical records of patients who underwent surgery for basic-type exotropia and had been followed up for ≥ 2 years were retrospectively recruited. Patients with myopia and spherical equivalent (SE) < -1.0 diopters (D) were excluded. The patients were classified according to the SE: group H had a SE ≥  + 1.0 D, and group E had -1.0 ≤ SE <  + 1.0 D. The surgical success rate and sensory outcome were compared. Surgical success was defined as exodeviation ≤ 10 prism diopters (PD) and esodeviation ≤ 5 PD at 6 m fixation. Stereoacuity was measured using the Titmus Preschool Stereoacuity Test.

**Results:**

Seventy-five patients (24 males and 51 females, mean age 5.1 ± 2.6 years, range 2.7–14.8) were included. The SE ranged from -0.9 to 4.4 and 21 patients were classified into group H and 54 into group E. The success rates were higher in group H than in group E during the entire follow-up period, but the differences were significant only at the final examination. At the final follow-up, 11 of the 21 (52.4%) patients in group H and 15 of the 54 (27.7%) in group E maintained successful alignment, whereas 10 (47.6%) and 38 (70.4%) patients exhibited recurrence. Overcorrection was exhibited in one (1.9%) patient in group E. Sensory results were comparable between the groups. The follow-up period did not differ between the two groups. The survival analysis showed no difference in the surgical results between the two groups.

**Conclusions:**

Surgery for basic-type intermittent exotropia resulted in superior outcomes in patients with hyperopia compared to those with emmetropia.

## Background

The success rate of exotropia surgery is likely to decrease over time. More than one operation is needed to obtain stable ocular alignment because of the high recurrence rate of surgery [1]. Various preoperative factors, including postoperative overcorrection, age at surgery, lateral incomitancey, amblyopia or anisometropia, and the degree of sensory binocularity, reportedly affect the recurrence of exotropia [2–8]. Rosenbaum and Santiago demonstrated that in patients with exotropia and hyperopia, exotropia could be undercorrected if the surgery was performed after measuring angle of deviation without refractive correction [9]. Additionally, it should be considered that refractive error may affect visual development in children. There are diverse opinions on whether refractive error and its correction affect the outcome of exotropia surgery. Both myopia and hyperopia have been reported to be good prognostic or risk factors [10, 11]. Conversely, studies have also reported that a small amount of refractive error does not affect the surgical outcome [12].

The effect of refractive error on exotropia surgery remains controversial, and this study aimed to investigate the effect of hyperopia on the long-term surgical outcomes of basic-type exotropia.

## Methods

### Patient recruitment

The Institutional Review Board of our hospital approved the study, and all medical procedures followed the tenets of the Declaration of Helsinki.

The medical records of patients with exotropia who underwent surgery by one surgeon (HYC) and were followed up for ≥ 2 years were retrospectively reviewed. Only basic-type exotropias with ≤ 10 prism diopters (PD) differences in the deviation angle at near and distance were included. Only patients with spherical equivalent (SE) greater than -1.0 were included in the study, and patients with anisometropia were excluded. Patients with any ophthalmological or neurological abnormality other than strabismus, paralytic or restrictive strabismus, or a history of previous ophthalmic surgery were excluded.

### Preoperative assessment

Using the prism and alternative cover tests, the deviation angle was measured using a target at 6 m (distance) and 1/3 m (near). After a 30-min patch test, the re-measurement of the strabismus with an additional + 3.0 D sphere lens over each eye was performed when the deviation angle at a distance was larger than that at near fixation. For patients wearing glasses, measurements were taken while wearing the glasses. Combined vertical strabismus and A or V patterns were evaluated, and version and duction were evaluated simultaneously. Near stereoacuity was measured using the Titmus Preschool Stereoacuity Test (Stereo Optical Co., Inc., Chicago, IL, USA). Refractive errors were determined by cycloplegic refraction. After instilling 3 drops of 1% cyclopentolate hydrochloride every 5 min, manual refraction was performed after 30 min. Anisometropia was defined as a spherical or cylindrical difference of at least 2.0 D between the eyes. Patients with a difference of two or more lines of visual acuity in each eye or subnormal visual acuity in their age group were considered to have amblyopia, and children with amblyopia were treated with occlusion therapy. Patients with hyperopia of > 2.00 D, astigmatism of > 1.5 D, or myopia at any range were prescribed glasses before a final surgical decision was made.

### Surgical intervention

Bilateral lateral rectus recession, unilateral lateral rectus recession, medial rectus resection, or unilateral lateral rectus recession was performed by a single surgeon (HYC). Surgery was performed if tropia was present for > 50% of the time or if there was deterioration in the frequency or magnitude of exotropia. All surgeries were performed under general anaesthesia. Patients without a dominant eye or amblyopia underwent bilateral lateral rectus recession, whereas others underwent unilateral lateral rectus recession combined with medial rectus resection. A unilateral lateral rectus muscle was performed if the deviation at 6 m was less than 20 PD. The number of surgeries was determined based on the Table 1 could be modified according to the surgeon’s experience. The target correction amount was determined based on the largest angle of deviation during distance fixation. Postoperative examinations were performed one week after surgery and followed up every 6 months.

**Table1:** Surgical dose

Ocular deviation (Prism diopters)	ULR recession (mm)	BLR recession (mm)	Recession and resection	
			**LR recession (mm)**	**MR resection (mm)**
16	8			
18	9			
20	10	5	5	4
25		6	6	5
30		7	7	6

*ULR* unilateral lateral rectus, *BLR* bilateral lateral rectus, *LR* lateral rectus, *MR* medial rectus

### Data analysis

The patients were allocated to one of two groups based on the preoperative SE. The one with the larger SE between the two eyes was taken: greater than or equal to + 1.0 Diopters (D) as group H (hyperopia group) and between -1.0 D and + 1.0 D as group E (emmetropia group).

Surgical success was defined as exodeviation ≤ 10 PD and esodeviation ≤ 5 PD at distance fixation at 1 and 2 years and the final examination postoperatively. All surgical outcomes were assessed using the first procedure. Recurrence was defined as > 10 PD of exotropia, and overcorrection was defined as > 5 PD of esotropia. Recurrence was defined as a reoperation due to recurrence. Stereoacuity ≤ 60 s of arc was considered indicative of a normal value. The determination to achieve normal stereoacuity was based on the last examination. The preoperative characteristics were compared between groups H and E. The proportions of patients in each group who achieved surgical success and normal stereoacuity were compared. Survival analysis for surgical success was also performed.

Quantitative data were described as means and standard deviations and were compared between the two groups using the Mann–Whitney U test. Categorical variables were described as distribution frequencies and analysed using Fisher’s exact or chi-square tests. All statistical analyses were performed using SPSS for Windows (version 21.0; SPSS, Chicago, IL, USA). Statistical significance was set at *P* < 0.05.

## Results

We included 75 patients (24 males and 51 females, mean age 5.1 ± 2.6 years, range 2.7–14.8) and divided into 21 in group H and 54 in group E. The two groups did not differ in terms of age and sex. The preoperative characteristics were not different except SE. Surgical methods did not differ between the two groups (Table 2). The surgical success rates were higher in group H than in group E at 1 and 2 years and the final examination; however, the differences were statistically significant only at the final examination. At the final follow-up, 11 of the 21 (52.4%) patients in group H and 15 of the 54 (27.7%) in group E maintained successful alignment, whereas 10 (47.6%) and 38 (70.4%) patients, respectively, exhibited recurrence. Overcorrection was observed in one (1.9%) patient in group E (Table 3)**.** Reoperation due to recurrence was noted in 25 patients (7 [33%] in group H and 18 [67%] in group E). The postoperative follow-up period did not differ between the two groups (58 months in group H vs 50 months in group E).

**Table 2:** Preoperative characteristics of the patients

	**Group H**	**Group E**	***P*-value**
Number of patients	21	54	
Sex (M:F)	9:12	15:39	0.163
Age at surgery (years, mean ± SD,(range))	4.8 ± 2.0 (2.7–8.9)	5.3 ± 2.8 (1.7–14.8)	0.672
Spherical equivalent (diopters, mean ± SD, (range))			
OD	1.2 ± 1.0 (0.0—4.4)	0.0 ± 0.5 (-0.9 – 0.9)	< 0.001
OS	1.0 ± 1.0(0 – 3.6)	-0.16 ± 0.80 (-0.9—0.9)	< 0.001
Visual acuity (logMAR, mean ± SD (range)			
OD	0.2 ± 0.2 (0.0 – 0.5)	1.1 ± 0.1 (0.0 – 0.5)	0.563
OS	0.2 ± 0.2 (0.0 – 0.8)	0.1 ± 0.1 (0.0 – 0.5)	0.488
Preoperative deviation (prism diopters, mean ± SD (range))			
At Distance	26.8 ± 8.0 (14—45)	28.3 ± 7.3 (16 – 45)	0.886
At Near	27.3 ± 9.4 (14—50)	29.4 ± 7.5 (16 – 45)	0.814
Associated strabismus (Number of the patients, %)			
Inferior oblique overaction	8 (38.1)	26 (48.1)	0.300
Dissociated vertical deviation	0 (0)	2 (3.7)	0.510
Combined vertical deviation	8 (38.1)	13 (24.1)	0.176
Type of surgery (Number of the patients, %)			0.134
BLR recession	11 (52.4)	39 (72.2)	
R&R	3 (14.3)	8 (14.8)	
ULR recession	7 (33.3)	7 (13.0)	
Follow-up period (months, mean ± SD, (range))	58.23 ± 29.91 (12 – 120)	50.17 ± 20.05 (18 – 92)	0.785

*SD* standard deviation, *BLR* bilateral lateral rectus, *R&R* unilateral lateral rectus recession—medial rectus resection, *ULR* unilateral lateral rectus

**Table 3:** Surgical outcomes of the two study groups

**Postoperative period**		**Group H** **n. of the patients (%)**	**Group E** **n. of the patients (%)**	***P*-value**
1 year	success	16 (76.2)	31 (57.4)	0.105
	Recurrence	5 (23.8)	21 (38.9)	
	overcorrection	0 (0)	2 (3.7)	
2 years	Success	13 (61.9)	25 (46.3)	0.169
	Recurrence	8 (38.1)	27 (50.0)	
	overcorrection	0 (0)	2 (3.7)	
Final examination	Success	11(52.4)	15(27.7)	0.040
	Recurrence	10(47.6)	38(70.4)	
	overcorrection	0(0)	1(1.9)	

**Fig. 1:**
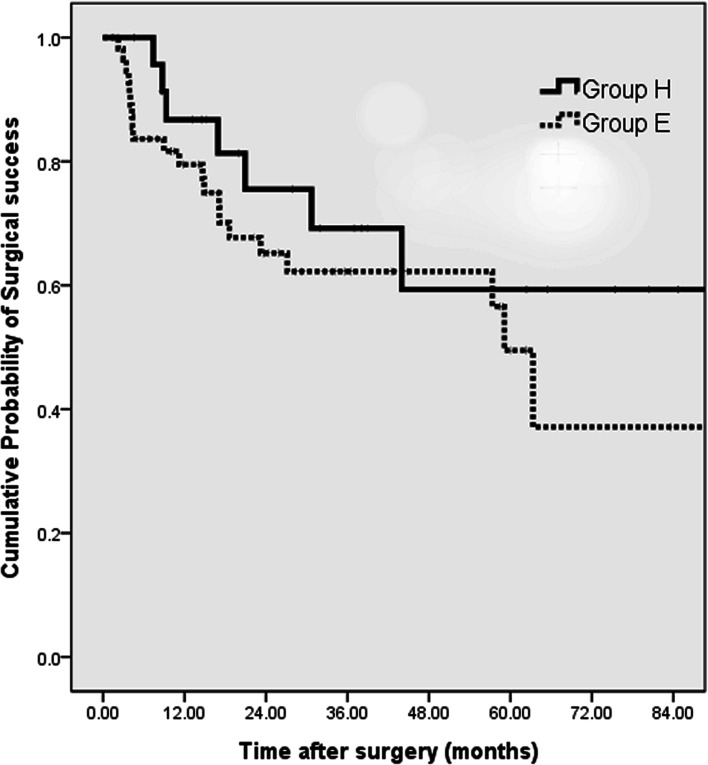
Kaplan–Meier survival analysis for surgical failure of exotropia in patients with hyperopia (Group H) and with emmetropia (Group E). The cumulative probabilities of surgical success in the two groups were not significantly different

Using Kaplan–Meier analysis, the estimated mean time to recurrence was 63.5 months in group H and 53.0 months in group E, indicating that the patients in group H maintained success for a longer time (Fig. 1).

All patients were checked for stereoacuity during the last examination. Normal stereoacuity was present in 19 (90.5%) patients in group H and 50 (92.6%) in group E, which was not significantly different between the two groups (*p* = 0.542, Fisher’s exact test). Among the 38 patients who underwent the stereoacuity test before surgery (10 in group H and 28 in group E), stationary or improved stereopsis (abnormal to normal) was found in all patients in group H, and 27 in group E. Decreased stereopsis (normal to abnormal) was found in one patient in group E (Table 4). This patient underwent a second surgery for recurrence and showed recurrence again at the final examination.

**Table 4:** Changes in stereoacuity in the two study groups

Stereoacuity	Group H number of patients (%)	Group E number of patients (%)
**Conducted in 38 patients**		
Preoperative		
Normal	1(10.0)	8(28.6)
Abnormal	9(90.0)	20(71.4)
At final examination		
Normal	7(70.0)	25(89.3)
Abnormal	3(30.0)	3(10.7)
Improvement	9(90.0)	25(89.3)
Stationary	1(10.0)	2(7.1)
Deterioration	0(0)	1(3.6)

Improvement; increased stereoacuity; Stationary: no change of stereoacuity; Deterioriation: decreased stereoacuity

Surgical complications, including limited ocular movement, symptomatic diplopia, or new-onset amblyopia, were not observed in any patient. However, one patient in group E exhibited sustained consecutive esotropia lasting 2 years, and the patient maintained good stereoacuity during the follow-up period. None of the patients had undergone surgery for consecutive esotropia.

## Discussion

In the present study, the success rate of exotropia surgery was not different at 1 and 2 years postoperatively in the two groups, according to the presence of hyperopia. Still, the differences were significant at the final examination (52.4% vs. 27.7%). The sensory outcome was comparable between the two groups.

Whether the refractive error is a risk factor for exotropia surgery is still debatable. Some investigators have demonstrated that refractive error is unrelated to the surgical prognosis of intermittent exotropia [13, 14]. Conversely, Kim et al. [11] reported superior surgical outcomes in hyperopic and myopic patients with intermittent exotropia than in patients with emmetropia in both motor and sensory aspects. He regarded hyperopia as a good prognostic factor in intermittent exotropia surgery because of younger age, longer follow-up period, and poor preoperative stereopsis in hyperopic patients.

The effects of fusional and accommodative convergence should be considered when prescribing hyperopic glasses to patients with exotropia. Correcting refractive errors might result in better control of the deviation because subnormal clarity in vision may promote impaired fusion and facilitate a manifest deviation [9]. Resolutions of exotropia after spectacle correction of moderate-to-severe hyperopia have been reported [15]. This would occur because fusional convergence improved with spectacles, and the patients had a relatively low accommodative convergence over accommodation ratio. Conversely, hyperopic correction in children with exodeviation should be carefully considered for fear of worsening exotropia [16]. It has been recommended that hyperopia of < 2.0 D should not be corrected in children with exodeviation because correcting any hyperopic refractive error will decrease the demand for accommodative convergence and thus increase both the frequency and the size of the exodeviation. Chung et al. [17] reported that some exotropia patients with moderate hyperopia demonstrated an increase in deviation after spectacle correction that was more pronounced in patients with pure hyperopia than in patients with hyperopic astigmatism or amblyopia. However, spectacle correction is still necessary to permit normal visual development in patients with amblyogenic degrees of hyperopia; therefore, partial or full spectacle correction may be prescribed. Consequently, hypo-correction of hyperopia as a non-operative treatment for exotropia that does not affect visual acuity may induce accommodative convergence, contributing to the long-term outcome of exotropia surgery.

A similar proportion of the patients in the two study groups achieved normal stereoacuity. It was reported that children with SE > 3.0 D were associated with a significantly reduced stereoacuity [18]. In our study, among the patients who could examine stereoacuity pre- and postoperatively, more patients in the hyperopia group were likely to show abnormal stereoacuity before surgery (90% vs. 71.4%). However, most patients showed improved or steady stereoacuity during the final examination. Additionally, there was no difference in the best-corrected visual acuity between the two groups before surgery. Taken together, there was no difference in preoperative visual function between the two groups, which may not have significantly affected the surgical results. Our study had some limitations. First, we did not evaluate axial length. Increasing the axial length of the globe can explain the importance of the myopic refractive state in determining surgical outcomes and its significance in response to surgery. Gezer et al. [8] reported an indirect relationship between refractive error and postoperative deviation. Small variations in the radius of the eyeball can significantly affect the number of surgeries required to correct horizontal strabismus [19]. The axial length of the two groups may help identify the causal relationship between refractive error and the outcome of surgery more precisely. Second, we needed to assess whether the success rates improved with multiple surgeries. In this study, we considered a case of reoperation as recurrence and did not include it in the range of success. An analysis of patients who underwent reoperation should be considered in future studies. Third, changes in refractive errors during the postoperative follow-up period were not analysed.

## Conclusion

Patients with hyperopia showed superior surgical outcomes than patients with emmetropia.

## Data Availability

All data was included in the manuscript.
